# Multimorbidity patterns as predictors of sleeping medication use: a population-based study in women in Southern Brazil

**DOI:** 10.1590/1980-549720240056

**Published:** 2024-12-09

**Authors:** Marina Luiza Grudginski de Oliveira, Michele Gabriela Schmidt, Jaqueline Stürmer, Débora Luiza Franken, Juvenal Soares Dias da Costa, Maria Teresa Anselmo Olinto, Vera Maria Vieira Paniz

**Affiliations:** I Universidade do Vale do Rio dos Sinos, Postgraduate Programme in Collective Health – São Leopoldo (RS), Brazil.; II Universidade Federal de Pelotas, Faculty of Medicine, Department of Social Medicine – Pelotas (RS), Brazil.; III Universidade Federal do Rio Grande do Sul, Faculty of Medicine – Porto Alegre (RS), Brazil.

**Keywords:** Multimorbidity, Multiple chronic conditions, Pharmaceutical sleep aids, Women, Multimorbidade, Múltiplas afecções crônicas, Medicamentos para dormir, Mulheres

## Abstract

**Objective:**

To explore the relationship between different patterns of multimorbidity and the use of sleeping medications in women.

**Methods:**

Population-based cross-sectional study with 1,128 women (aged 20–69 years) in Southern Brazil. Data on sleeping medications were obtained from the question "Do you take/use any medication to be able to sleep?" and identified by the Anatomical Therapeutic and Chemical classification. Multimorbidity patterns were derived by the Principal Component Analysis of 26 chronic conditions and two obesity parameters (≥30 kg/m^2^; ≥40 kg/m^2^). The association was analyzed by Poisson regression with robust variance using different adjustment models, stratified by age.

**Results:**

Three multimorbidity patterns were derived: cardiometabolic, endocrine-articular, and psychosomatic. Age stratification showed a change in effect in the relationship investigated. Women under 45 years and high score of cardiometabolic and endocrine-articular patterns were about twice as likely to use sleeping medications [prevalence ratio (PR) 1.85, 95% confidence interval (CI) 1.09–3.12; PR 2.04, 95%CI 1.18–3.51, respectively]. Those with psychosomatic pattern were around five times more likely [PR 4.91, 95%CI 3.00–8.04].

**Conclusions:**

The study provided the first evidence on the association researched and demonstrated that young women (<45 years) with a high score of the identified patterns are up to five times more likely to use sleeping medications, configuring early use. This unprecedented finding suggests the need for greater health promotion for young adults and actions to raise awareness about risks and the clear indication of the use of sleeping medications.

## RESUMO

**Objetivo:**

Explorar a relação entre diferentes padrões de multimorbidade e o uso de medicamentos para dormir em mulheres.

**Métodos:**

Estudo transversal de base populacional com 1.128 mulheres (20–69 anos) no Sul do Brasil. Dados sobre medicamentos para dormir foram obtidos por meio da pergunta "Você toma/usa algum medicamento para conseguir dormir?" e identificados pela Classificação Anatômica Terapêutica e Química. Os padrões de multimorbidade foram derivados pela Análise de Componentes Principais de 26 condições crônicas e dois parâmetros de obesidade (≥30 kg/m^2^; ≥40 kg/m^2^). A associação foi analisada por regressão de Poisson com variância robusta utilizando diferentes modelos de ajuste, estratificados por idade.

**Resultados:**

Foram derivados três padrões de multimorbidade: cardiometabólico, endócrino-articular e psicossomático. A estratificação etária mostrou mudança de efeito na relação investigada. Mulheres com idade inferior a 45 anos e alto escore nos padrões cardiometabólico e endócrino-articular tiveram cerca de duas vezes mais probabilidade de usar medicamentos para dormir [razão de prevalência (RP) 1,85, intervalo de confiança (IC) 95% 1,09–3,12; RP 2,04, IC95% 1,18–3,51, respectivamente]. Aquelas com padrão psicossomático tiveram cerca de cinco vezes mais probabilidade (RP 4,91, IC95% 3,00–8,04).

**Conclusões:**

O estudo forneceu as primeiras evidências sobre a associação investigada e demonstrou que mulheres jovens (<45 anos) com alto escore dos padrões identificados têm até cinco vezes mais probabilidade de usar medicamentos para dormir, configurando uso precoce. Essa constatação inédita sugere a necessidade de maior promoção da saúde dos jovens e de ações de conscientização sobre os riscos e da indicação clara do uso de medicamentos para dormir.

## INTRODUCTION

The use of pharmacotherapy for sleep disorders has increased considerably in recent years. Sleep-aiding psychotropic drugs have been widely prescribed, and the duration of treatment often exceeds the recommended period of up to three months^1^. Epidemiological studies seeking to elucidate the factors associated with increased prescription and use of sleep aids are still inconclusive^2^. Sleep disorders are a frequent complaint in public health but predisposing conditions are not well established^3^.

Additionally, sleeping disorders are more prevalent among women and individuals with chronic diseases^4^. Women, due to their own reproductive and hormonal characteristics, experience sleep differently through^5^ the use of sleep-aided medications, with a prevalence range observed from 10–28%^2,6^. The findings reported a linear increase with age, but more significant in the transition from reproductive to non-reproductive life due to the poorer quality of sleep during this period^5,7^. Moreover, the literature reveals a direct relationship between the number of chronic conditions and sleep problems^8^. It also indicates that specific conditions such as chronic pain and cardiometabolic disorders, alone or in combination, may be predictors of sleep disorders and potentially contributors to the use of sleep medications^8–10^. Recent researches indicate that the presence of two or more chronic conditions in the same individual, defined as multimorbidity, may increase the probability of the occurrence of these disorders^8,10,11^.

Studies have shown that individuals with multimorbidity are more likely to be prescribed hypnotic/anxiolytic medications^9,12^. Common mental disorders (CMD) represent one of the most frequent coexisting diagnoses in different combinations of chronic conditions^6^; however, the use of sleeping medications is strongly associated with multimorbidity among women, even in those without medical reports of CMD^2^.

Furthermore, in most investigations, obesity is a risk factor for multimorbidity; however, other studies suggest that it should be considered as a component condition of some combinations of chronic conditions^13,14^. A previous research conducted on women aged 20–69 years that investigated the effect of multimorbidity on sleeping medication use, considering obesity as an independent variable (risk factor or potential confounding factor) and a component of the multimorbidity outcome, found that in multimorbidity women (≥2; ≥3 chronic conditions), the probability of using these drugs was approximately double, even after adjustment for CMD^11^.

Thus, this study aimed to explore the relationship between different patterns of multimorbidity and the use of sleeping medications in adult women.

## METHODS

### Study design and target population

A population-based cross-sectional study was conducted in 2015 (from February to October) with a representative sample of women aged 20–69 years residents of the municipality of São Leopoldo, state of Rio Grande do Sul, Brazil. This study is part of a larger study that aimed to evaluate the living and health conditions of adult women. We included women in the age group of interest who lived in the selected sectors and households, except pregnant women.

### Sampling and data collection

The sample size was calculated based on the outcomes of interest. We chose the one that required a larger sample size (1,013 women), added 10% for eventual losses and refusals, and 15% to control for confounding factors in the data analysis, totaling 1,281 women. The sampling was probabilistic and performed in multiple stages. Initially, we estimated the average number of women per household in the municipality of São Leopoldo and their proportion in the age group of interest. A total of 371 census tracts in the urban area were classified in descending order according to residents’ monthly income. The sectors were numbered from 1–371, of which 45 were randomly selected and subsequently totaled 36 households in each sector. Then, the blocks and corners of each conglomerate were drawn to start the research. The households were designated according to the following rule: the corner indicated the starting point, always to the left of those facing the initial corner, the first house was selected for the study, skipping two houses and again selected the fourth house, and so on, until 36 households per sector were completed. Trained interviewers collected the data through the application of a standardized questionnaire previously tested in a pilot study. Quality control for data was applied to 10% of the participants.

### Variables analyzed

Data on the use of sleeping medications (dependent variable) were obtained through the question "Do you take/use any medication to get to sleep?". The name of the drugs, indication, and duration of use were also requested. Sample calculations were performed using the Epi Info 7.2.5.0 program (https://www.cdc.gov) with an estimated prevalence of 15%, margin of error of 2.5 percentage points, and design effect of 1.37, as detailed in a previous publication^11^.

To identify chronic conditions, we applied the report on the current consumption of medications of continuous use as a proxy^15,16^. It was determined according to the Anatomical Therapeutic and Chemical (ATC) classification: level 1, anatomical group N; and levels 2 and 3, therapeutic and chemical groups and the prescribed indications mentioned by the participants^17^. Ultimately, 26 chronic conditions were identified, with the most prevalents being: hypertension, CMDs, acid-related digestive disorders, dyslipidemia, thyroid diseases, diabetes mellitus, circulatory disorders, and chronic pain^18^.

Explanatory sociodemographic variables and possible confounding factors included: age (20–34, 35–44, 45–54, 55–69 years) analyzed as <45 and ≥45 years; self-reported skin color (white/other–black, yellow, parda, Indigenous, and others); marital status (having a partner or not); education in years of study (≥11, 8–10, 5–7, and ≤4 years); economic class (A+B, C, and D+E) according to the economic classification criteria of the Brazilian Association of Research Companies (ABEP, Associação Brasileira de Empresas de Pesquisa; https://www.abep.org/); household income per capita in quartiles (first ≤R$525.30, second R$525.31–869.00, third R$869.01–1547.00, and fourth ≥R$1547.01) corresponding to about two minimum wages, considering a national minimum wage of R$788.00, approximately U$245 at the time of the study; and occupation (employed/unemployed).

Behavioral variables were adequate consumption of fruits and vegetables (≥5 servings daily)^19^; alcohol consumption established based on frequency, type of beverage, and daily amount ingested, considering 15 g of ethanol/day as a cutoff point^20^; current smoker; and leisure-time physical activity, according to the International Physical Activity Questionnaire-Short Form (IPAQ-SF) categorized as active (≥150 min/week of moderate/vigorous physical activity) or inactive (<150 min/week)^21^. Health variables were body mass index (BMI), classified according to the criteria of the World Health Organization: (low weight <18.5 kg/m^2^, eutrophy 18.5–24.9 kg/m^2^, overweight 25.0–29.9 kg/m^2^, obesity ≥30.0 kg/m^2^, being Class I obesity 30.0–34.9 kg/m², Class II obesity 35.0–39.9 kg/m^2^, and Class III obesity ≥40 kg/m^2^)^22^; and CMD (absent <7 points and present (≥7 points), according to the score obtained through the Self-Reporting Questionnaire (SQR-20)^23^.

### Data analysis

Multimorbidity patterns were derived from two approaches using Principal Component Analysis (PCA). In the first, ten chronic conditions were considered (prevalence ≥2%)^18^. In the second, obesity was included (BMI≥30 kg/m^2^) as well as morbid obesity (BMI≥40 kg/m^2^)^11^. The factors obtained were rotated through orthogonal rotation (Varimax), which minimizes the number of variables with high factor loads in each factor, increasing the accuracy of the analysis, and ensuring non-correlation between the factors^24^. Because each chronic condition was coded as a dichotomous variable, the tetrachoric correlation matrix between all conditions was calculated^14^. The number of retained factors was based on components with eigenvalues >1 and a scree-plot test. Chronic conditions were considered loaded on one factor if they had an absolute correlation ≥0.3 with the factor^24^. Before defining the number of chronic conditions included in the PCA, Kaiser-Meyer-Olkin's (KMO) and Bartlett's sphericity tests were used to verify the applicability of the analysis. Factor scores were saved for each participant individually. Multimorbidity patterns were divided into tertiles based on their scores, categorized into low (first and second tertiles) and high (third tertile)^14^.

The univariate analysis described the sample, the bivariate analysis used Pearson's chi-square and linear trend tests, and the association between multimorbidity patterns and sleeping medication use was assessed through Poisson regression with robust variance. The crude and adjusted prevalence ratios (PRs) obtained were described with their respective 95% confidence intervals (95%CI). Variables with p≤0.20 in the crude analysis were considered potential confounds. The possible interaction between participants’ age and the main exposure was investigated using the Mantel-Haenszel^1,7^. A p<0.05 in the homogeneity test (M-H) defined the stratification of the analyses by age (<45 and ≥45 years). Multivariate analysis was performed according to different adjustment models: Model I (unadjusted PR), Model II (Model I + sociodemographic characteristics), Model III (Model II + behavioral variables), and Model IV (Model III + health variables), and significance level p<0.05. The diagnosis of each model for each level of analysis (II, III, and IV) was performed using the poisgof command. The statistically significant value (p<0.05) of the test indicates that the model is inappropriate. The detected values ranged from 0.6107 to 0.9997. In sensitivity analyses, we calculated crude and adjusted PRs for sleeping medications use according to the multimorbidity patterns generated by both approaches and additionally without stratification by age group. Statistical analyses were performed using Stata 13.0 (Stata Corp., College Station, Texas, USA).

The study was conducted following the Declaration of Helsinki guidelines and approved by the Research Ethics Committee of the University of Vale do Rio dos Sinos (CAAE 30872914.6.0000.5344, protocol 650.443). All participants provided written informed consent.

## RESULTS

The total number of women visited was 1,281, of which 1,128 were interviewed, representing losses or refusals of 11.9%. The participants were 43.4 years on average, standard deviation (SD) ±13.4 years. Most reported working (56.0%), 18.4% were smokers, and 66.8% consumed alcoholic beverages. Overweight/obesity were identified in two-thirds of the sample (66.0%), and CMD in approximately 40.0%. Sleeping medications were used by 14.3% (95%CI 12.2–16.3) of participants, and showed a direct linear association with age, inverse association with schooling, and were more prevalent among women who did not work, who did not consume alcoholic beverages, were former smokers, were overweight/obese, and had CMD (Table 1).

**Table 1:** Sample distribution and prevalence of sleep medication use according to sociodemographic, behavioral, and health characteristics in women in Southern Brazil (n=1,128).

Variables*		n (%)	Use of sleep medications	
			95%CI	p-value*
Age (years)				
	20–34	344 (30.5)	5.2 (2.9–7.6)	<0.001^a^
	35–44	264 (23.4)	13.3 (9.1–17.4)	
	45–54	247 (21.9)	18.2 (13.4–23.1)	
	55–69	273 (24.2)	23.1 (18.0–28.1)	
Skin color				
	White	840 (74.5)	15.2 (12.8–17.7)	0.114^b^
	Other (black, brown, Indigenous, yellow, and others)	288 (25.5)	11.5 (7.8–15.2)	
Marital status				
	Not having a partner	408 (36.2)	15.2 (11.7–18.7)	0.505^b^
	Having a partner	720 (63.8)	13.8 (11.2–16.3)	
Education (years)				
	≥11	470 (41.7)	9.4 (6.7–12.0)	<0.001^a^
	8–10	199 (17.7)	14.1 (9.2–18.9)	
	5–7	253 (22.5)	17.8 (13.0–22.5)	
	0–4	204 (18.1)	21.6 (15.9–27.3)	
Economic class (ABEP)				
	A + B	390 (34.8)	13.9 (10.4–17.3)	0.640^a^
	C	596 (53.1)	15.6 (12.7–18.5)	
	D + E	136 (12.1)	10.3 (5.1–15.5)	
Household income per capita (quartiles)				
	I (low)	273 (25.0)	11.7 (7.9–15.6)	0.345^a^
	II	273 (25.0)	17.2 (12.7–21.7)	
	III	273 (25.0)	11.7 (7.9–15.6)	
	IV (high)	272 (24.9)	16.5 (12.1–21.0)	
Occupation				
	Employed	654 (58.1)	9.5 (7.2–11.7)	<0.001^b^
	Unemployed	472 (41.9)	20.8 (17.1–24.4)	
Consumption of fruits and vegetables				
	Adequate (≥5 servings daily)	492 (43.7)	15.0 (11.9–18.2)	0.531^b^
	Inadequate (<5 servings daily)	634 (56.3)	13.7 (11.0–16.4)	
Alcohol consumption				
	Does not consume	346 (33.2)	19.9 (15.7–24.2)	<0.001^b^
	Consumes	697 (66.8)	10.2 (7.9–12.4)	
Smoking status				
	Non-smoker	661 (58.6)	12.3 (9.7–14.8)	0.023^b^
	Former smoker	259 (23.0)	19.3 (14.5–24.1)	
	Current smoker	208 (18.4)	14.4 (9.6–19.2)	
Physical activity				
	Active	162 (14.4)	17.3 (11.4–23.2)	0.236^b^
	Inactive	966 (85.6)	13.8 (11.6–15.9)	
Body mass index†				
	Low weight/Eutrophy	380 (33.9)	10.8 (7.7–13.9)	0.107^a^
	Overweight	373 (33.2)	15.6 (11.9–19.2)	
	Obesity	369 (32.9)	16.5 (12.7–20.3)	
	Class I	205 (18.3)	18.5 (13.2–23.9)	
	Class II	94 (8.4)	12.8 (5.9–19.6)	
	Class III	70 (6.2)	15.7 (7.0–24.5)	
Common mental disorders‡				
	Absence	677 (60.1)	7.1 (5.2–9.0)	<0.001^b^
	Presence	450 (39.9)	25.1 (21.1–29.1)	

ABEP: Brazilian Association of Research Companies; 95%CI: 95% confidence interval;

* Maximum values were 85 for "alcohol consumption";

† Body mass index (BMI): Low Weight/Eutrophy <25.0 kg/m^2^, Overweight 25.0–29.9 kg/m^2^, Obesity ≥30 kg/m^2^: Class I obesity 30.0–34.9 kg/m^2^, Class II 35–39.9 kg/m^2^, and Class III obesity ≥40 kg/m^2^;

‡ Common mental disorders (CMD): Self-Reporting Questionnaire (SQR) score ≥7.

a p-value of the chi-square test for linear trend;

b p-value of the chi-square test for heterogeneity of proportions.

Three patterns of multimorbidity were derived by PCA that explained 45.5% of the total variance: cardiometabolic (dyslipidemia, circulatory disorders, arterial hypertension, and diabetes), endocrine-articular (thyroid diseases, osteoporosis/osteopenia, and rheumatic diseases), and psychosomatic (chronic pain, CMD, and acid-related digestive disorders). The KMO coefficient was 0.720 with p≤0.001 for the Bartlett's test, suggesting the adequacy of the analysis^24^. Among the ten chronic conditions included, only acid-related digestive disorders were saturated in >1 pattern. Cardiometabolic pattern had the highest percentage (21.9%) of explained variance (Table 2). In PCA, which included obesity, a fourth pattern was derived, explaining 9.4% of the total variance (Supplementary Table 1). The KMO coefficient was 0.711 with p≤0.001 for Bartlett's test.

**Table 2:** Factorial loading matrix^a^ for the multimorbidity patterns found derived with ten chronic conditions in women aged 20–69 years, São Leopoldo (RS), Brazil, 2015 (n=1,128).

	Factor/Pattern		
	Cardiometabolic	Endocrine-articular	Psychosomatic
Dyslipidemia	**0.7417**	0.1732	-0.0539
Circulatory disorders	**0.6658**	-0.1415	0.0285
Arterial hypertension	**0.6657**	0.1308	0.1646
Diabetes	**0.6336**	-0.0456	0.0401
Thyroid diseases	-0.0570	**0.7018**	0.0072
Osteoporosis/Osteopenia	0.0780	**0.6150**	-0.0210
Rheumatic diseases	0.0461	**0.5944**	0.1258
Chronic pain	0.0441	0.0082	**0.7106**
Common mental disorders	0.0122	0.0257	**0.6987**
Acid-related digestive disorder	**0.3848**	0.2290	**0.4045**
Explained variance (%)^b^	**21.9**	**13.1**	**10.5**

a Factor loadings indicate the strength of association between each variable and each factor, with a factor loading of <0.3 (non-bold loadings) generally considered to be weak;

b The percentage of total variance accounted by all factors is 45.5%.

Table 3 shows that a high score of the identified multimorbidity patterns was verified in older women, with less schooling, unemployed, with adequate consumption of fruits and vegetables, who did not consume alcoholic beverages, were former smokers, and obese. High scores for cardiometabolic and psychosomatic patterns were identified in women with CMD and those on sleeping medications.

**Table 3:** Prevalence of low and high scores of multimorbidity patterns according to sociodemographic, behavioral, and health characteristics in women in Southern Brazil (n=1,128).

Variables		Multimorbidity patterns									
		n	Cardiometabolic			Endocrine-articular			Psychosomatic		
			Low	High	p-value	Low	High	p-value	Low	High	p-value
			%	%		%	%		%	%	
**Prevalence**			69.4	30.6		74.3	25.7		69.5	30.5	
Age (years)											
	20–34	344	92.7	7.3	<0.001^a^	92.4	7.6	<0.001^a^	89.0	11.0	<0.001^a^
	35–44	264	82.2	17.8		81.1	18.9		71.6	28.4	
	45–54	247	61.9	38.1		69.6	30.4		58.7	41.3	
	55–69	273	34.4	65.6		49.1	50.9		52.8	47.2	
Skin color											
	White	840	71.0	29.0	0.056^b^	75.1	24.9	0.277^b^	69.4	30.6	0.902^b^
	Other (black, brown, Indigenous, yellow, and others)	288	64.9	35.1		71.9	28.1		69.8	30.2	
Marital status											
	Not having a partner	408	71.1	28.9	0.361^b^	76.5	23.5	0.207^b^	72.1	27.9	0.161^b^
	Having a partner	720	68.5	31.5		73.1	26.9		68.1	31.9	
Education (years)											
	≥11	470	81.3	18.7	<0.001^a^	80.6	19.4	<0.001^a^	78.5	21.5	<0.001^a^
	8–10	199	74.9	25.1		77.4	22.6		75.4	24.6	
	5–7	253	60.1	39.9		68.4	31.6		58.9	41.1	
	0–4	204	49.0	51.0		64.2	35.8		55.9	44.1	
Economic class (ABEP)											
	A + B	390	75.6	24.4	0.014^a^	76.9	23.8	0.567^a^	73.3	26.7	0.197^a^
	C	596	65.6	34.4		71.6	28.4		66.3	33.7	
	D + E	136	69.1	30.9		77.9	22.1		71.3	28.7	
Household income per capita (quartiles)											
	I (low)	273	71.8	28.2	0.932^a^	79.5	20.5	0.016^a^	73.6	26.4	0.394^a^
	II	273	69.6	30.4		77.7	22.3		69.6	30.4	
	III	273	63.4	36.6		67.0	33.0		63.7	36.3	
	IV (high)	272	73.5	26.5		73.5	26.5		72.1	27.9	
Occupation											
	Employed	654	80.3	19.7	<0.001^b^	81.0	19.0	<0.001^b^	76.6	23.4	<0.001^b^
	Unemployed	472	54.2	45.8		64.8	35.2		59.7	40.3	
Consumption of fruits and vegetables											
	Adequate (≥5 servings daily)	492	60.2	39.8	<0.001^b^	65.9	80.8	<0.001^a^	64.0	36.0	<0.001^b^
	Inadequate (<5 servings daily)	634	76.5	23.5		34.1	19.2		73.7	26.3	
Alcohol consumption											
	Does not consume	346	57.5	42.5	<0.001^b^	67.6	32.4	<0.001^b^	59.5	40.5	<0.001^b^
	Consumes	697	77.9	22.1		78.8	21.2		75.9	24.1	
Smoking status											
	Non-smoker	661	71.9	28.1	0.001^b^	75.8	24.2	<0.001^b^	71.9	28.1	0.041^b^
	Former smoker	259	59.9	40.1		65.3	34.7		63.3	36.7	
	Current Smoker	208	73.6	26.4		80.8	19.2		69.7	30.3	
Physical activity											
	Active	162	73.5	26.5	0.228^b^	71.0	29.0	0.299^b^	71.0	29.0	0.658^b^
	Inactive	966	68.7	31.3		74.9	25.2		69.3	30.7	
Body mass index*											
	Low weight/Eutrophy	380	86.8	13.2	<0.001^a^	86.3	13.7	<0.001^a^	80.8	19.2	<0.001^a^
	Overweight	373	70.2	29.8		73.5	26.5		68.9	31.1	
	Obesity	369	50.7	49.3		63.1	36.9		58.5	41.5	
	Class I	205	54.6	45.4		69.3	30.7		61.0	39.0	
	Class II	94	48.9	51.1		54.3	45.7		59.6	40.4	
	Class III	70	41.4	58.6		57.1	42.9		50.0	50.0	
Common mental disorders†											
	Absence	677	73.3	26.7	0.001^b^	74.9	25.1	0.616^b^	77.0	23.0	<0.001^b^
	Presence	450	63.8	36.2		73.6	26.4		58.4	41.6	
Use of sleep medications											
	No	967	72.8	27.2	<0.001^b^	76.0	24.0	0.001^b^	75.5	24.5	<0.001^b^
	Yes	161	49.1	50.9		64.0	36.0		33.5	66.5	

ABEP: Brazilian Association of Research Companies.

* Low weight/Eutrophy <25 kg/m^2^, Overweight 25.0–29.9 kg/m^2^; Obesity ≥30 kg/m^2^: Class I obesity 30.0–34.9 kg/m^2^, Class II obesity 35.0–39.9 kg/m^2^, and Class III obesity ≥40 kg/m^2^;

† Common mental disorders (CMD): Self-Reporting Questionnaire (SRQ) ≥7.

a p-value of the chi-square test for linear trend;

b p-value of the chi-square test for heterogeneity of proportions.

In Table 4, after adjusting for confounding factors, there was an increased probability of sleeping medication use in the presence of a high score of multimorbidity patterns identified among younger women (<45 years). A high cardiometabolic and endocrine-articular multimorbidity pattern score doubled the probability of sleeping medication use among women in this age group (PR 1.85, 95%CI 1.09–3.12; PR 2.04, 95%CI 1.18–3.51, respectively). The high score of the psychosomatic multimorbidity pattern increased the probability of sleeping medication consumption by about five times in women aged <45 years and twice among women aged ≥45 years (PR 4.91, 95%CI 3.00–8.04; PR 1.96, 95%CI 1.30–2.94, respectively).

**Table 4:** Crude and adjusted sleep medication use analyses according to low and high scores of multimorbidity patterns derived with ten chronic conditions in the different adjustment models according to age groups <45 years and ≥45 years in women in Southern Brazil (n=1,128).

		Model I		Model II		Model III		Model IV	
		<45	≥45	<45	≥45	<45	≥45	<45	≥45
		PR (95%CI)	PR (95%CI)	PR (95%CI)	PR (95%CI)	PR (95%CI)	PR (95%CI)	PR (95%CI)	PR (95%CI)
Cardiometabolic									
	p-value*	0.003	0.009	0.002	0.063	0.002	0.281	0.022	0.399
	Low	1	1	1	1	1	1	1	1
	High	2.42 (1.36–4.30)	1.60 (1.12–2.28)	2.42 (1.39–4.22)	1.42 (0.98–2.07)	2.38 (1.37–4.13)	1.24 (0.84–1.84)	1.85 (1.09–3.12)	1.18 (0.80–1.74)
Endocrine-articular									
	p-value*	0.001	0.922	0.001	0.624	0.001	0.520	0.008	0.697
	Low	1	1	1	1	1	1	1	1
	High	2.51 (1.43–4.41)	0.98 (0.70–1.38)	2.53 (1.45–4.42)	0.92 (0.65–1.30)	2.48 (1.42–4.33)	0.88 (0.60–1.29)	2.08 (1.21–3.58)	0.93 (0.65–1.33)
Psychosomatic									
	p-value*	<0.001	<0.001	<0.001	<0.001	<0.001	<0.001	<0.001	0.001
	Low	1	1	1	1	1	1	1	1
	High	6.67 (4.00–11.13)	2.84 (1.96–4.12)	5.98 (3.63–9.86)	2.60 (1.80–3.77)	5.98 (3.65–9.80)	2.41 (1.61–3.60)	4.91 (3.00–8.04)	1.96 (1.30–2.94)

PR: prevalence ratio; 95%CI: 95% confidence interval; Model I: unadjusted prevalence ratio; Model II: Model I + sociodemographic variables; Model III: Model II + behavioral variable; Model IV: Model III + health variables.

* Variables associated with use of sleep medications with a p≤0.20 were kept in the model as potential confounding factors.

All sensitivity analyses showed results similar to our main results and provided evidence of the detected interaction (Supplementary Tables 2-5).

## DISCUSSION

This study investigated the relationship between patterns of multimorbidity and sleeping medication use in a representative sample of women aged 20–69 years. The consumption of sleeping medications was verified in approximately 15% of women, and three patterns of multimorbidity were identified: cardiometabolic, endocrine-articular, and psychosomatic. The association between high cardiometabolic and endocrine-articular multimorbidity pattern scores and outcome was modified by age. Younger women (<45 years) were twice as likely to use sleeping medications but in the next age group (≥45 years), this association was not verified. For the high psychosomatic pattern score, the probability of using sleeping medications doubled in this age group and was five times greater in younger women, even after adjusting for CMD.

The prevalence of sleeping medication use was higher than those identified in studies carried out in Europe^6^ and lower than that observed in women aged ≥25 years in Spain (18.1%)^7^. However, in a study conducted in Canada with women ≥18 years, a prevalence of 14.6% was found^1^. In Brazil, a national survey observed the prevalence of 10.4% in this age group^25^. However, the frequency of sleeping medication use has increased in recent decades among women. A cohort study in Australia with a 20–year segment observed that consumption quadrupled over this period^26^. Furthermore, there is a linear increase with age, especially in the age group ≥45 years^1,7^, which is consistent with this study which found that sleeping medication use doubled among women in this age group.

The patterns of cardiometabolic, endocrine-articular, and psychosomatic multimorbidity identified corroborated the literature^13,14,27^. However, methodological differences in pattern derivation, target population, and amplitude of the age groups investigated may explain some specificities. In this sense, the chronic conditions that derived the endocrine-articular and psychosomatic patterns in the present study also composed a unique pattern in a similar study^27^. Another aspect pertinent to the composition of the patterns involves the complex underlying mechanisms of multimorbidity and health determinants, such as psychosocial, behavioral, socioeconomic, and population-level intervention factors that reflect on the structure of health in different countries.

Considering the explanatory variables examined, the highest prevalence of patterns of multimorbidity and sleeping medication use was verified in older women, those with low schooling, unemployed, not consuming alcoholic beverages, with CMD, and obese, converging with the literature^6,13,25^. However, inconsistency has been identified in the association with smoking. Studies point to smoking as a predictor of sleeping medication use due to the stimulating effect of nicotine^7,25^. In our study, women who quit smoking were more likely to consume sleeping medications. Thus, it is considered relevant to investigate the duration of nicotine abstinence and the need for sleeping medications in future studies.

In this study, the relationship assessed was modified by the participants’ age. Although sleeping medication use has shown a direct linear association with age, the probability of use was higher among younger women with high scores for the identified patterns. Some authors suggest that chronic pain present in certain patterns, cardiovascular diseases, or dysregulated hormone levels negatively impact sleep and may affect the sleep-wake cycle^8,10^. Still, in premenopausal women thyroid disorders are common^28^ and the symptoms include inability to rest, agitation, and anxiety that lead to difficulty sleeping. It is plausible to think that the presence of this condition in the endocrine-articular pattern could lead to the consumption of these medications in younger women.

Our findings evidenced the high score of the psychosomatic pattern as an important predictor of sleeping medication use, especially in younger women. Evidence points toward an emerging increase in the prevalence of CMD among younger women, which could explain, at least in part, our results^12^. Chronic pain and acid-related digestive disorders also compose this pattern. The literature indicates a bidirectional relationship, permeated by biological and psychological factors, between CMD and chronic pain, which could interfere with sleep quality, leading to the consumption of sleeping medications^29^. It is important to note that the prescription of sleeping medications without elucidation of the predisposing conditions that permeate sleep disorders in multimorbid and polymedicated women exposes this population to harmful effects that increase mortality and burden health services. Finally, the presence of acid-related digestive disorders in this pattern of multimorbidity is hypothesized to be due to pharmacotherapy associated with chronic pain, including non-steroidal anti-inflammatory drugs related to injury to the stomach epithelium by inhibiting the protective factors of the gastric mucosa^30^.

Exploration of the role of obesity, either as a risk factor or chronic condition, in the construction of multimorbidity patterns is still controversial in the literature. In this sense, a previous study in this population observed twice the likelihood of using sleeping medications in case of multimorbidity (≥2; ≥3) including obesity as a chronic condition and risk factor; however, the patterns of multimorbidity were not investigated^11^. In the present study, when investigating obesity for the construction of patterns, the formation of a single pattern was observed regardless of the cutoff point used (≥30 kg/m^2^; ≥40 kg/m^2^). Thus, considering that obesity alters metabolism and generates an inflammatory process that contributes to the development of chronic health conditions^31^, it is plausible to think about the possible precursor role of obesity in multimorbidity patterns generation, i.e., obesity would indirectly influence the use of these medications.

Study limitations include the possibility of temporality bias inherent in cross-sectional studies and analyses based on self-reported data. However, the study considered the diagnoses of chronic conditions already under pharmacological treatment prescribed by a physician, validating the presence of these conditions. Regarding PCA, subjective decisions such as the definition of diseases^18^, cutoff point for factor loading^24^, and number of factors to be retained^24^ were consistent with the literature^14,24^.

To the best of our knowledge, this is the first study to investigate the relationship between multimorbidity patterns and sleeping medication use in a representative sample of women. Besides, it promoted a new contribution to the area. The strategy employed to identify chronic health conditions allowed the inclusion of an unlimited number of morbidities already established by drug use, rather than a predefined list, as observed in most available studies. Furthermore, the statistical method applied for the derivation of multimorbidity patterns does not establish a priori restrictions on the derivation of factors or the number of factors to be retained; it allows each variable to load more than one factor. The analysis of morbid obesity allowed the exploration of its effect on sleeping medication use, an aspect not previously investigated.

Although both sleeping medication use and multimorbidity patterns were more frequent in older women, our study demonstrated a higher probability of consumption among younger women (<45 years) with high scores for the identified multimorbidity patterns, suggesting that specific aspects of some combinations of chronic conditions can interfere with the quality of sleep. Given the increasing prevalence of multimorbidity and the consumption of sleeping medications at early ages, understanding the relationship between different patterns of multimorbidity and the use of these medications becomes important to guide the clinical management and rational indication of sleep aids, and to design strategies for the prevention and care of women with multimorbidity, especially younger women.

## Data Availability

The datasets generated and/or analyzed during the current study are available from the corresponding author on reasonable request.
